# Properties of Hyper-Elastic-Graded Triply Periodic Minimal Surfaces

**DOI:** 10.3390/polym15234475

**Published:** 2023-11-21

**Authors:** Christopher W. Haney, Hector R. Siller

**Affiliations:** Department of Mechanical Engineering, University of North Texas, 3940 N. Elm Str., Denton, TX 76207, USA; christopherhaney@my.unt.edu

**Keywords:** hyper-elastic, lattice structures, triply periodic minimal surfaces, functionally graded, energy absorption, cyclic compression

## Abstract

The mechanical behaviors of three distinct lattice structures—Diamond, Gyroid, and Schwarz—synthesized through vat polymerization, were meticulously analyzed. This study aimed to elucidate the intricacies of these structures in terms of their stress–strain responses, energy absorption, and recovery characteristics. Utilizing the described experiments and analytical approaches, it was discerned, via the described experimental and analytical procedure, that the AM lattices showcased mechanical properties and stress–strain behaviors that notably surpassed theoretical predictions, pointing to substantial disparities between conventional models and experimental outcomes. The Diamond lattice displayed superior stiffness with higher average loading and unloading moduli and heightened energy absorption and dissipation rates, followed by the Gyroid and Schwarz lattices. The Schwarz lattice showed the most consistent mechanical response, while the Diamond and Gyroid showed capabilities of reaching larger strains and stresses. All uniaxial cyclic compressive tests were performed at room temperature with no dwell times. The efficacy of hyper-elastic-graded models significantly outperformed projections offered by traditional Ashby–Gibson models, emphasizing the need for more refined models to accurately delineate the behaviors of additively manufactured lattices in advanced engineering applications.

## 1. Introduction

Because of their ability to create flexible, lightweight, and deformable structures, soft materials have received recent attention amongst researchers. These materials’ low Young’s moduli at room temperature and strong elongation characteristics make them easily deformable by thermal and mechanical loads. This has allowed a range of novel approaches used in the fabrication of parts paired with additive manufacturing (AM) to create hyper-elastic, flexible, durable, and tunable structures [1,2,3,4,5,6]. Recent literature has proved the immense benefits of AM and its ability to rapidly reproduce complex geometries in a variety of materials compared to traditional manufacturing processes [7,8,9,10]. The most recent trend with lattices has enabled further weight and waste reductions while offering a key incentive for topology optimization (TO) and functional geometry applications [11,12,13]. Iterations of lattices have attracted interest for their vibrational damping [14], osteointegration in orthopedic implants [8,15,16], strength-to-weight ratios in aerospace applications [17], and energy absorption capabilities [18,19,20].

**Figure 1:**
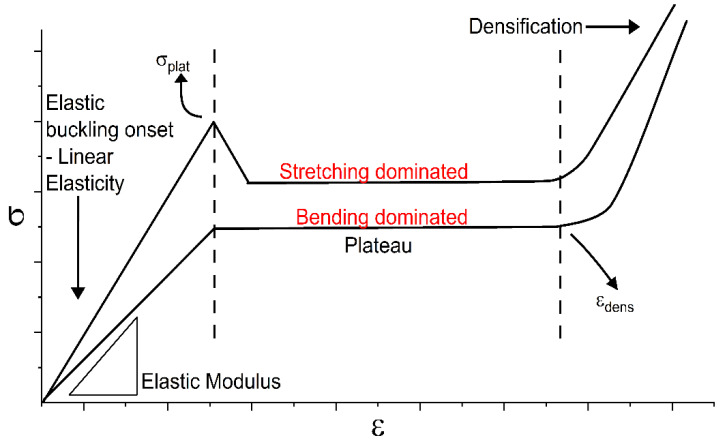
Stretching versus bending behavior with defined plateau and densification regime.

Scientists characterize lattices by their relative density (ρ*/ρ_s_) [21] or volume fraction to explore the performance of various cell types and materials according to preferred porosity and fabrication process constraints. Designs can then be tailored to specific application requirements, a focal point of functionally graded materials (FGM) [14,22,23,24,25]. Conventional processes for FGM fabrication tend to suffer geometry limitations. However, AM has shown ease in generating complex internal and external features with higher resolution, efficiency, and minimal waste for uniform Triply Periodic Minimal Surface structures (TPMS) [26,27,28]. This has allowed for the opportunity to investigate previously developed minimal surfaces and the effects grading has on the performance compared to predictions using Ashby–Gibson models. These models are formulated based on beam theory and mechanics to simplify the formulations for key parameters like stiffness, plateau stress, and densification. These mathematically controlled surfaces can extend and repeat in the x, y, and z dimensions, having large surface areas, high tortuosity, better mass transfer, pore interconnectivity, and thermal conductivity [29]. In addition, TPMSs possess a zero mean curvature and are free from self-intersections, reducing potential print errors caused by overhangs that may plague designs. The literature has demonstrated that cell shape has a significant impact on deformation and failure processes, providing an opportunity to adjust designs to avoid undesired failure modes during loadings. Cell designs with walls normal to the loading direction tend to provide better stiffness but give rise to two dominating behaviors [25]. Bending and stretching dominated structures, commonly described in cellular solids, as illustrated in Figure 1, with stretching dominated structures exceeding their bending counterparts in stiffness and peak stress [30]. Previous studies have examined the distinctions between sheet and skeleton Triply Periodic Minimal Surfaces (TPMSs). These investigations revealed that although both types undergo similar deformation processes, sheet TPMSs stand out for their enhanced ability to regulate relative density, chiefly exhibiting stretching dominated behaviors [23,31,32,33]. Comparing the elastic modulus, yield strength, stress–strain distribution, and energy characteristics of rigid materials is a common literary practice, with limited contributors on graded soft structures and the recoverable deformation properties [25,34].

In this paper, vat polymerization is used to design and fabricate three hyper-elastic iterations of functionally graded TPMS structures. Weight scaling is used to assess printing and manufacturing accuracy. The cell wall thickness is graded in the z direction, resulting in a primary density transition near the base. Compressive cyclic loadings were employed in experimental testing to examine the Young’s modulus, plateau stress, densification strain, and energy characteristics across repeated cycles. Finally, the mechanisms of energy absorption are investigated. Here, the normalized energy absorption values (W/E_s_) are calculated through integration of the σ-ε curves and normalization via the material Young’s modulus. This value is plotted against values for normalized peak stress (σ_p_/E_s_) on a logarithmic scale. The energy absorption capacities of various structures are examined, as well as the energy absorption rates under different compression phases.

## 2. Method and Materials

### 2.1. TPMS Development

#### 2.1.1. TPMS Solid–Void Boundary Definitions

Three iterations of CAD TPMS were constructed. Small arrays were selected to explore feasibility and initial characteristics before scaling for future practical applications. This approach aides in validating the methodology and manufacturing process. By using small-scale arrays, the material usage, manufacturing, and testing processes can be streamlined prior to using more representative volumes. Solid–void boundary equations, such as those defining Diamond and Schwarz geometries, are mathematical expressions used to delineate the shift between material and empty space within a given lattice structure. In this study, these equations were procured from 3.31.2.0, nTopology, Inc., New York, NY, USA, a software platform for advanced generative design and optimized for developing high-performance parts, allowing precise control over the geometric configurations of the designed structures. The following equations describe the TPMS surfaces aligned with the cartesian x, y, and z directions employed by the CAD modeling software used to construct the geometries (3.31.2.0, nTopology, New York, NY, USA):

1. Schoen Gyroid

(1) $$U_{G}={\left(S_{X}C_{y}+S_{Y}C_{z}+S_{z}C_{X}\right)}^{m}-t_{m}$$

2. Schwarz Diamond

(2) $$U_{D}={\left(S_{x}S_{y}S_{z}+S_{x}C_{y}C_{z}+C_{x}S_{y}C_{z}+C_{x}C_{y}S_{z}\right)}^{m}-t_{m}$$

3. Schwarz Primitive

(3) $$U_{p}={\left(C_{x}+C_{y}+C_{z}\right)}^{m}-t_{m}$$

where shorthand notation is used:

(4) $$S_{i}=\sin\left(k_{i}\frac{i}{L_{i}}\right)$$

(5) $$C_{i}=\cos\left(k_{i}\frac{i}{L_{i}}\right)$$

(6) $$k_{i}=2\pi n_{i}$$

The variable *k_i_* is the periodicity of the lattice function, with *i = x*, *y*, *z* representing directions and *n_i_* representing the number of cell repetitions in those directions. *C_i_* and *S_i_* represent sine and cosine functions, with *i* again representing the different x, y, and z directions and *L_i_* indicating the absolute size of the lattice structure. The variable *t* is used to regulate the lattice’s volume percent. Lattice structures with an arbitrary number of cells and relative densities can be fabricated using Schwarz Primitive, Diamond, and Schoen Gyroid models. The variable *m* is used to switch between network (*m* = 1) and matrix (*m* = 2) phases, also known as skeleton and sheet TPMS. The Diamond and Gyroid lattices are members of a class of minimum surface equations that lack reflection or rotational symmetry on their primary axis. Equations with just an even term (cosine) exhibit this behavior. Regions with *U_i_ ≥* 0 are considered solids, whereas regions less than zero are defined as voids. Changing *t* shifts the solid–void border, resulting in a larger or smaller region to affect the relative density. Cell size and wall thickness changes will be used to produce functionally graded lattice structures with a target relative density of 30% or 0.3.

#### 2.1.2. Modeling and Fabrication

The CAD (computer-aided design) modeling software nTopology was utilized to create three iterations of 16 mm cubes filled with TPMS structures of specified cell sizes. To ensure a controlled wall thickness throughout the structure, a refined voxel grid of 0.1 mm was used during the CAD process, which allows for high-quality mesh creation without the need for further refinement. The ramping function was applied to regulate the wall thickness, smoothly transitioning from a fully dense state at the base (3.5–4 mm to ensure solidity) to about 0.7 mm (printer’s minimum capability) at +4 mm in the z-direction. The samples were exported to STL (Standard Tessellation Language) format, maintaining the integrity of the detailed TPMS structures due to the chosen voxel grid approach.

Samples were oriented vertically in the Formlabs 3+ printer using Preform software, employing Formlabs Elastic 50A resin (3.14.0, Formlabs, Boston, MA, USA) for vat polymerization. This vertical orientation was maintained to ensure consistent layer build-up during the printing process and reduced the need for additional support material. The 50A resin, whose mechanical properties are cataloged in Appendix A, was cured using a 250 mW laser with an 85 μm spot size. The Form 3+ printer is capable of printing with a 25–300 μm Z resolution and a 25 μm XY resolution. The resolution of the prints will be controlled by the laser spot size and the material layer thickness (XY: 85 μm, Z: 100 μm). This careful arrangement ensured that the solid base volume firmly adhered to the build plate, which is critical for the stability of the structure.

The cell dimensions for the Gyroid, Diamond, and Schwarz TPMS structures were tailored to maintain the target relative density of 0.3. Lattice model volumes, based on the material density provided by the manufacturer (ρ_50a_ = 0.00102 g/mm^3^), and were anticipated to weigh around 1.25 g. Post-fabrication, the samples underwent a dual stage washing in 99% isopropyl alcohol (IPA) for ten minutes each, followed by a twenty-minute post-cure at 60 °C under 1.25 mW/cm^2^ of 405 nm light, a procedure that enhances the mechanical properties by promoting interlayer adhesion [7] and polymer cross-linking.

### 2.2. Mechanical Tests and Characterization

Electric vernier calipers were employed to quantify the geometric features of the nine cubes, and a calibrated scale was utilized to weigh each sample. The values were rounded to the nearest hundredth place. Cyclic uniaxial compression experiments were conducted at a speed of 5 mm/min until a strain limit of ε ≅ 70% was reached, given by the 11 mm stroke limit set for testing. No dwell intervals were employed during the transfer between loading and unloading for the three cycles (N_total_ = 3, N = 1, 2, 3). The samples were compressed using a Shimadzu Universal Testing Machine (Shimadzu, Kyoto, Japan) at room temperature T_amb_ ≈ 21 °C, with a 10 kN load cell. The parts were placed in the middle region of the bottom base plate for testing. Each test was performed once per sample, where the original, undeformed length and cross-sectional area were used for the stress and strain calculations of each cycle. The displacement and reaction forces were determined by the movement of the upper compressive head and accompanied Trapezium software (1.4.0. Trapezium, Shimadzu, Kyoto, Japan). These values were then used to determine the engineering stress–strain (*σ*-*ε*) diagrams. The engineering *σ*-*ε* diagrams were evaluated to determine the following:

- the experimental Young’s modulus and plateau stress related to predicted values by theoretical models;
- energy absorption, recovery, and dissipation extracted from *σ*-*ε* diagrams;
- and normalized energy absorption per unit volume (W/E_s_) vs. normalized peak stress (σ_p_/E_s_).

The elastic modulus was estimated using a linear fitting to the identified elastic regime using the first 200 data points. This is discernible prior to the yielding and subsequent constant plateau stress. According to Hooke’s law, the slope of the line gives us the elastic modulus. The energy absorption and strain energy recovery values are determined by applying Ashby–Gibson’s open-cell deformation theory and taking the integral of the *σ*-*ε* functions up to a chosen strain interval, *ε_eng_*. This takes the form of the following:

(7) $$\int_{0}^{\varepsilon }\sigma \left(\varepsilon \right)d\varepsilon$$

In this case, *ε* denotes the given strain, and *σ* corresponds to the compressive stress. The amount of energy absorbed can be calculated by integrating the loading curve, and the amount of strain energy recovered can be calculated by using the unloading curve’s integral. The difference between these two values represents the energy dissipation and is identified as the shaded region between the loading and unloading curves. The normalized energy absorption for elastic open-cell materials can be ascertained by taking the stepwise integral and using the solid material modulus (*E_s_*) that comprises the lattice for normalization. This takes the form of the following:

(8) $$\frac{1}{E_{s}}\int_{0}^{\varepsilon }\sigma \left(\varepsilon \right)d\varepsilon$$

The energy absorbed per unit volume between different lattices for a given relative density per normalized peak stress can be compared using this method. The energy absorption rates under discrete compressive stages are discussed in the following results section, where a more detailed equation is provided.

## 3. Results

**Figure 2:**
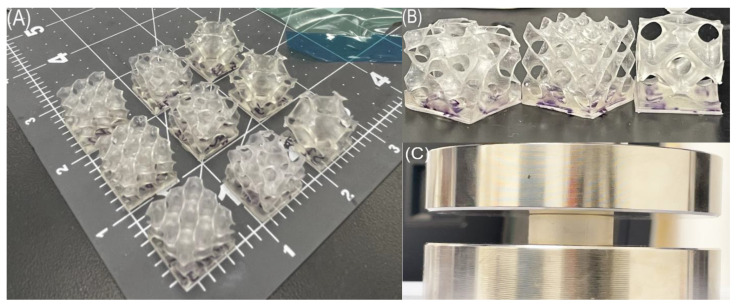
(**A**) Each iteration of printed sample on an inch scale, (**B**) a close-up of individual Gyroid, Diamond, and Schwarz lattices (from left to right) in 50 A 16 mm graded cubes, (**C**) test stand image of tested solid sample in uniaxial compression.

Figure 2 shows the printed parts after post-processing on an inch scale. One Gyroid sample had two visible tears in its walls after post-processing. The *σ*-*ε* diagrams and energy properties of cellular solid samples are investigated in the following sections, using cyclic compressive testing at a constant strain rate. The *σ*-*ε* curves for all of the models began with a linear elastic region given up to the point of yield where individual cell walls buckle. After reaching yield, stress reaches a semi-constant value, corresponding to cell layer collapse via cell wall elastic bending, reaching a long plateau. Following cell collapse, opposing walls begin to touch and the densification regime commences. This final regime is recognized as the area following the plateau where the stress starts to increase quickly. Observed data from compressive testing were converted into engineering stress–strain graphs by using Equations (9) and (10) where the engineering stress ($\sigma_{eng})$ is determined, using the applied load *F* = force and *A*_0_ = initial cross-sectional area of the following sample:

(9) $$\sigma_{eng}=\frac{F}{A_{0}}$$

(10) $$\varepsilon_{eng}=\frac{l-l_{0}}{l_{0}}$$

In a solid material with no or minimal porosity, the cross-sectional area is the area of the solid section. However, in porous materials, the cross-sectional area includes both the solid material and the voids. For a TPMS sample or any porous material, when calculating the cross-sectional area, both the solid part and the voids within that cross-section need to be considered. The actual solid area will be less than the total cross-sectional area due to the presence of voids. The engineering strain ($\varepsilon_{eng})$ is determined by taking the difference in length during testing divided by the initial length. From these diagrams, a noticeable large variation of Gyroid 1 (G1) compared to Gyroid 2 and 3 (G2 and G3) *σ*-*ε* data exists because of the tears. Variations in the samples’ relative density are indicated by the value in the top left. These deviations likely result from z-thickness in the transition zone trapping resin and extra material added at the base for printability. Additionally, minor internal defects and resin deposits in cavities near transition zones might be present. The densification strain according to the Ashby–Gibson models was also plotted for reference in the *σ*-*ε* diagrams to further compare predictions and experimental results. The approximate onset of densification can be determined as follows:

(11) εDens=1−1.4ρ*ρs

where ρ*ρs is the relative density of the sample determined from the ratio of lattice mass to volume over the density of the material shown in the top left.

### 3.1. Compressive Mechanical Properties

**Figure 3:**
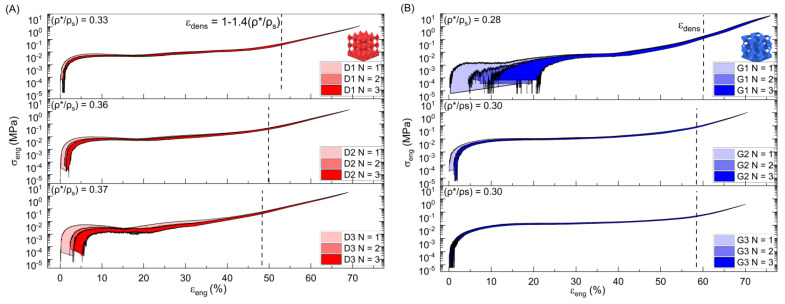

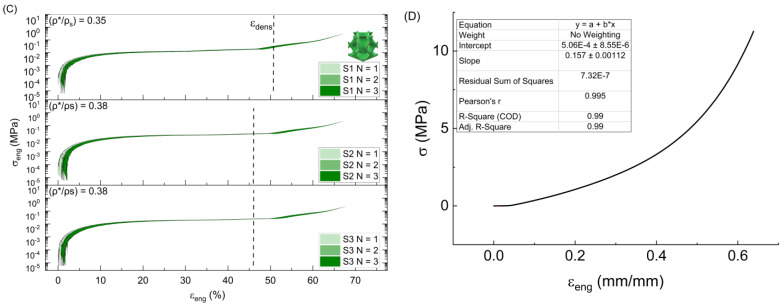
Cyclic compressive σ-ε data with theoretical densification strain for (**A**) Diamond, (**B**) Gyroid, (**C**) Schwarz, and (**D**) the compressive testing of a solid 50 A sample with linear fitting.

The *σ*-*ε* (stress–strain) diagrams in Figure 3 depicts cyclic compressive testing from experimentation using constant strain control. “Dx” stands for Diamond, where “x” = 1, 2, and 3 and represents each individual printed sample. Gyroid and Schwarz are represented by the “Gx” and “Sx” notation, respectively, while “N” represents the compressive cycle number.

All the stress-strain graphs have a linear elastic region at the beginning and ending of the compressive cycles used to establish the loading and unloading of the Young’s modulus. Minor variations in *σ*-*ε* diagrams were noticed for the lattices after each successive loading cycle where the final unloading strain did not reach 0 exactly but ended near *ε* = 1 mm/mm. This behavior indicates the presence of some negligible residual stress from testing which may become more apparent at higher cycles. Each compressive cycle begins at a null stress condition before advancing to the maximum stress value indicated by the maximal strain. The loading is then removed at the same rate back to a null stress condition. Because of this, for all of the tests, (*σ*_max_/*σ*_min_) → R = 0 and indicates that the loading regime is characterized by an absence of tensile stresses in each cycle.

No extensometer was used due to the concern of contact influencing cell collapse and densification behavior, experiment samples undergoing large strain deformation, and expected non-uniform behavior. The predictive moduli for each lattice structure using only the relative density ρ*ρs and solid material modulus $\left(E_{s}\right)$ was determined and shown compared to the Ashby–Gibson equation:

(12) E*=Esρ*ρS11+ρ*ρs

**Figure 4:**
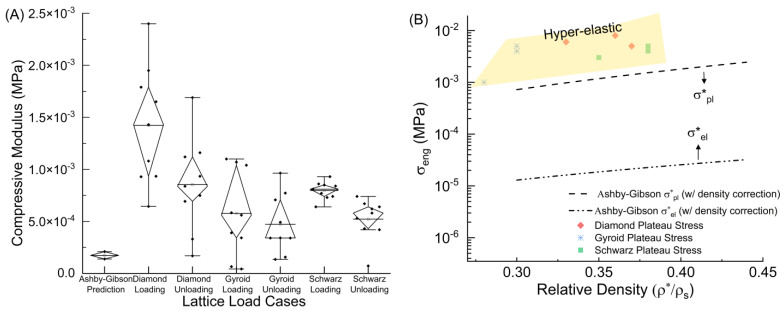
(**A**) Elastic modulus results and Ashby–Gibson prediction, (**B**) Ashby–Gibson elastic and plastic plateau stress predictions for relative densities > 0.3.

Figure 4A demonstrates the much higher modulus values obtained from experimental results compared to the predictions. The Gyroid and Diamond lattices present slight dips that correspond to individual layer collapse, whereas the Schwarz displays a consistent plateau transition. Some residual strains were noticed for the samples, but no stress stiffening was observed. G1 had large residual strains due to tears present in some cell walls, causing a shift in the general deformation and lattice recovery mechanism. A modulus of 0.158 MPa was determined for the solid sample. This was then used to determine the theoretical plateau stress (σ*), Young’s modulus (E*), and densification strain (*ε_d_*) by using the determined relative density and solid material modulus (*E_s_*). The corresponding equations were used to determine the upper ((ρ*/*ρ_s_*) = 0.44) and lower ((ρ*/*ρ_s_*) = 0.28) limits displayed in Figure 4B for the theoretical elastic and plastic moduli [21]. The minimum relative density (0.30) was selected based on the print with the lowest relative density, excluding G1 because of the torn cell walls, while the maximum (0.44) was chosen to guarantee the acquisition of a comprehensive set of relevant data.

The Diamond lattice had the highest average loading and unloading modulus (1.42 × 10^−3^ MPa, 8.5 × 10^−4^ Mpa), followed by the Schwarz (7.97 × 10^−4^ Mpa, 5.22 × 10^−4^ Mpa), and finally the Gyroid (5.77 × 10^−4^ Mpa, 4.72 × 10^−4^ Mpa). This correlates with previous research that also found the Diamond to have the best stiffness of these tested samples for high strains [32,33,35]. All of the tested graded structures yielded modulus values much higher than the models predicted. The Diamond lattices produced plateau stresses, σ*, between 0.005–0.008 Mpa, while the Gyroid produced values between 0.001–0.005 Mpa, and the Schwarz generated values between 0.003–0.005 Mpa, higher than the elastic and plastic predictions for the material, using the equations:

(13) σEL *≈ 0.03ESρ*ρs21+ρ*ρs1∕22

(14) σPL *≈ 0.23σYSρ*ρs321+ρ*ρs1∕2

outlined in Figure 4B for those relative density ranges [21]. In Ashby–Gibson’s equations, the elastic plateau stress was based on the solid material’s stiffness, suitable for modeling reversible, elastic deformation. Conversely, the plastic plateau stress used the solid material’s yield stress, relevant for predicting permanent, plastic deformation. This distinction ensured each equation accurately reflected the material’s behavior under different stress conditions. The theoretical plateau stress was calculated for the outlined relative densities for comparison. The bottom dash-dot-dot line represents the projected plateau stress for elastic materials, whereas the top dash line represents the predicted plateau stress for plastic materials with both containing a density adjustment, required for relative densities greater than 0.3 or 30%. It is clearly established here that the 3D-printed samples performed far better than traditional models expected for plateau stress [21]. Elastic 50 A functionally graded models attained values greater than the projected plastic plateau stress while preserving the capacity to fully recover from deformations.

The efficacy of the hyper-elastic-graded models substantially surpasses the estimations rendered by the Ashby–Gibson paradigms, underscoring a pronounced divergence between theoretical predictions and empirical outcomes. It is apparent that extant models, predominantly tailored to conventional foams, falter in delineating the intricate behaviors of engineered materials in additive manufacturing (AM). The foundational simplifications and assumptions embraced by these models remain discordant with the diverse realities of materials synthesized through additive methodologies, thus fostering incongruities between prognostications, and exhibited results.

These paradigms’ foundational assumptions and simplifications do not align well with the intricate realities of additively manufactured materials, resulting in noticeable variances between anticipated and actual outcomes. Given the precision needed for design and application in advanced engineering fields, there is a pressing necessity for the creation of enhanced, more inclusive models that can accurately represent the diverse nature and behaviors of latticed AM materials. These should consider factors like print orientation, the inclusion of polymer fillers or additives, the formation of interlayer boundaries during printing, and the compositions and cross-linking capabilities, each significantly impacting material performance. For example, optimizing print orientation can improve surface resolution and reduce layer lines, thereby decreasing potential stress concentrators during loading.

Furthermore, the selected lattices, known for their sophisticated structures, provide improved surface area-to-volume ratios, isotropy, effective stress and load distribution, and better energy dissipation. To achieve heightened accuracy, it is imperative to embrace more sophisticated modeling methodologies and conduct thorough experimental analyses, taking into consideration the material behavior, fabrication procedures, and lattice configurations. Adopting more refined and nuanced modeling approaches is crucial to understanding the diverse variables in AM, ensuring the development of materials that meet the rigorous requirements of modern engineering.

### 3.2. Energy Absorption Properties and Relative Error

The subsequent sections are directed towards the themes of energy recovery, absorption, dissipation, and error in the context of the studied material and designs. A comprehensive exploration of energy recovery mechanisms is essential, as it provides insight into the efficacy of the model and material in converting and reusing energy. Furthermore, an in-depth understanding of energy properties provides a valuable lens through which resilience and longevity under operational conditions can be assessed. Equally crucial is the precise quantification and analyses of error, ensuring the reliability and validity of the results observed and contributing to the refinement of existing models and methodologies.

#### 3.2.1. Absorption, Recovery, and Dissipation

The area or difference between the loading and unloading areas reflects the energy dissipated, whereas the area under the individual loading–unloading curves indicates the absorbed energy and recoverable strain energy; energy dissipation can then be defined as such:

(15) $$\left[\int \sigma {\left(\varepsilon \right)}_{load}\mathrm{d}\varepsilon -\int \sigma {\left(\varepsilon \right)}_{unload}\mathrm{d}\varepsilon \right]$$

**Figure 5:**
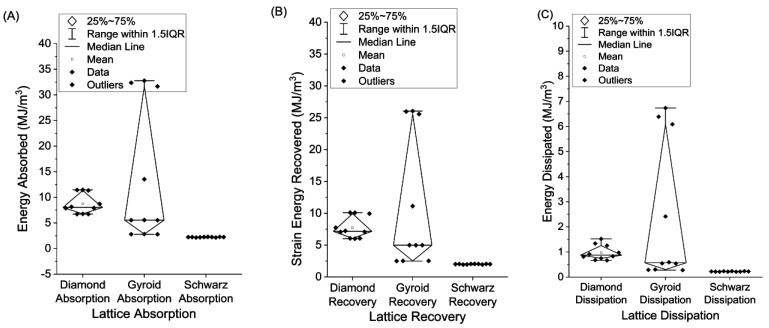
(**A**) Energy absorption, (**B**) energy recovery, (**C**) energy dissipated for Diamond, Gyroid, and Schwarz 16 mm graded cubes.

The integrated values obtained for all loading and unloading cycles per lattice for the given absorption, recovery, and dissipation are shown in Figure 5. The Diamond plots show the middle half of the data. The top and bottom bands represent the 75^th^ and 25^th^ percentiles, respectively. The Interquartile Range (IQR) spans between these two quartiles. A hollow square indicates the mean, while the median is marked by a horizontal line within each Diamond. The data exhibit some skewness, which the Gyroid sample clearly demonstrates, due to the unequally spaced distances from the upper and lower bands. Any points outside of the IQR are thought to be suspected outliers. The whiskers at the ends of the bands represent the maximum and minimum values or up to a maximum of 1.5 times the IQR.

**Figure 6:**
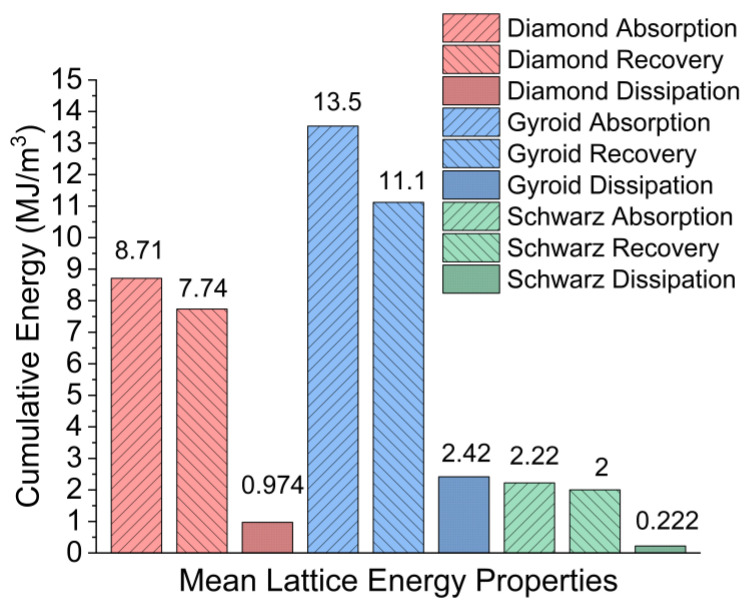
Mean energy absorption, recovery, and dissipation values for Diamond, Gyroid, and Schwarz lattices after three cycles of compression.

The energy absorption has a small spread for the Diamond, a larger spread for the Gyroid due to G1, and almost no spread for the Schwarz. This is generally true across the board for absorption, recovery, and dissipation rates of the printed samples. The Gyroid on average, shown in Figure 6, absorbed the most energy (13.54 MJ/m^3^), recovered the most strain energy (11.12 MJ/m^3^), and dissipated the most energy (2.42 MJ/m^3^), with G1 being the major element contributing towards that. During compression, the Diamond lattice absorbed 8.71 J/m^3^, recovered 7.74 MJ/m^3^ from strain energy, and dissipated 0.97 MJ/m^3^. The Schwarz produced the lowest but most consistent energy absorption (2.23 MJ/m^3^), strain energy recovery (2.00 MJ/m^3^), and dissipation (0.22 MJ/m^3^) values. Excluding the influence from G1, the adjusted Gyroid values for energy absorption, strain energy recovery, and energy dissipation become 4.17 MJ/m^3^, 3.75 MJ/m^3^, and 0.42 MJ/m^3^, which were values less than those produced by the Diamond samples, but still higher than the tested Schwarz.

#### 3.2.2. Normalized Energy Absorption

Cellular solids are used for their advantageous qualities in impact protection, and one of the key criteria in their application is their energy absorption behavior. By combining the data integral of *σ*-*ε* data for several lattices, we may learn more about the relationship between the absorption of energy per unit of volume (*W_v_*) and engineering strain (*ε_eng_*) by using normalized energy (*W*/*E_s_*) graphs where:

(16) $$\frac{W_{v}}{E_{s}}=\frac{\int \sigma \left(\varepsilon \right)\mathrm{d}\varepsilon }{E_{s}}\approx \sum_{i=1}^{n}\frac{\sigma_{i-1}+\sigma_{i}}{2}\left(\varepsilon_{i}-\varepsilon_{i-1}\right)$$

**Figure 7:**
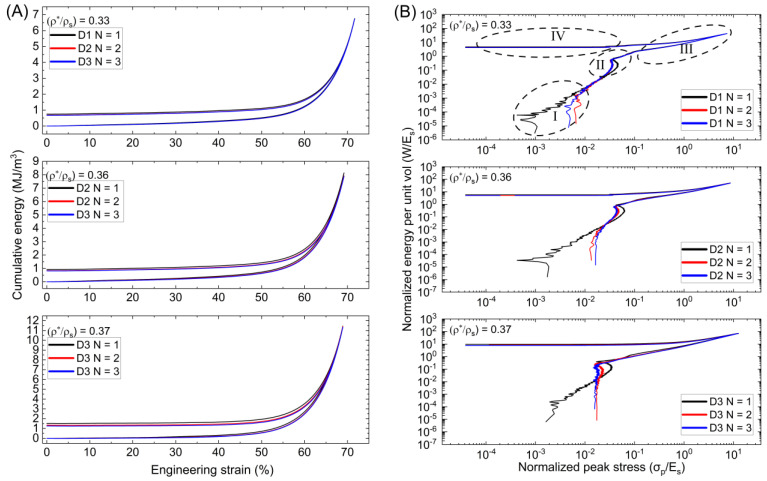
(**A**) Cumulative energy per strain and (**B**) Normalized energy per normalized peak stress for Diamond lattices. “I” denotes the linear elastic region, where the material deforms elastically and stress is proportional to strain. “II” represents the plateau or yield region, indicating the point at which the material yields and deforms at a nearly constant stress level. “III” corresponds to the densification region, where the material’s opposite cell walls touch, leading to a sharp increase in stress. “IV” is the recovery phase, which refers to the ability of the material to recover its shape after the removal of stress.

The previously collected energy data (*W_v_*) is normalized by the solid material modulus (*E_s_*) for every given strain during testing. Stress data are then normalized by (*E_s_*) to provide the relation between normalized peak stress (*σ_p_*/*E_s_*), and normalized energy absorbed per unit volume (*W*/*E_s_*) of a lattice, as illustrated in Figure 7. According to the *Wv*-*ε_eng_* curves, the energy absorbing rate for the graded Diamond lattice increases smoothly until it reaches a strain interval of 50%, before surging. Degradation and weakening are observed between each compressive loading and recovery. When the load is removed, the energy rates rapidly decrease from a peak of 6.74 MJ/m^3^, 8.14 MJ/m^3^, and 11.46 MJ/m^3^ for D1, D2, and D3, respectively, before reaching a steady state that lasts until the end of compressive testing. All of the Diamond samples exhibit similar behavior, with the D3 sample producing the highest peak energy values and D2 producing values slightly higher than D1. Ashby et al. [21,36] were the first to show how to streamline the correlations of successive compressive phases by plotting the cumulative normalized energy per unit of volume against the peak stress and applying logarithms to (*W*/*E_s_*) and (*σ_p_*/*E_s_*). The curve serves as an illustration of a lattice structure that absorbs the necessary force by reducing stress.

According to changes in slope, the curves are separated into four regions, with each region reflecting the energy characteristics associated with compressive stages. The first region describes the elastic buckling of the lattice with only a little quantity of energy absorbed during compression. The first cycle displays a Diamond lattice with a steadily ascending slope compared to the successive cycles, which, for D1, present the same slope as the initial cycle, but for D2 and D3, a more vertical slope is present. The initial normalized peak stress value for D1 shifts from (2.9 × 10^−5^) to (1.4 × 10^−4^) at the conclusion of testing for D1. All three samples exhibit this shift, corresponding to the strain hardening of the material after load application. Within region I, there are horizontal peaks that signify the collapse of individual cells before the collapse of cell layers. Region II shows the complete collapse of the structure with a significant increase in energy absorbed with minimal stress increase before sharply turning for the next regime. The structure begins to densify in area III, showing a steady slope toward the end of the loading cycle. The slope accelerates at a much more moderate rate as the stress increases. The strain energy recovery and unloading process is represented by region IV. The slope from peak (*W*/*E_s_*) and (*σ*/*E_s_*) inside this region gradually decreases to a constant value until the testing is complete. Sample D3 shows a more vertical slope, following compressive stress at greater relative density. The distance between the shoulder points and strain recovery phases is closer for relative densities of 0.33 and 0.36 than at 0.37 for the Diamond sample. It is also clear that the D3 sample has a different behavior leading to the should-point than the D2 and D1 samples.

**Figure 8:**
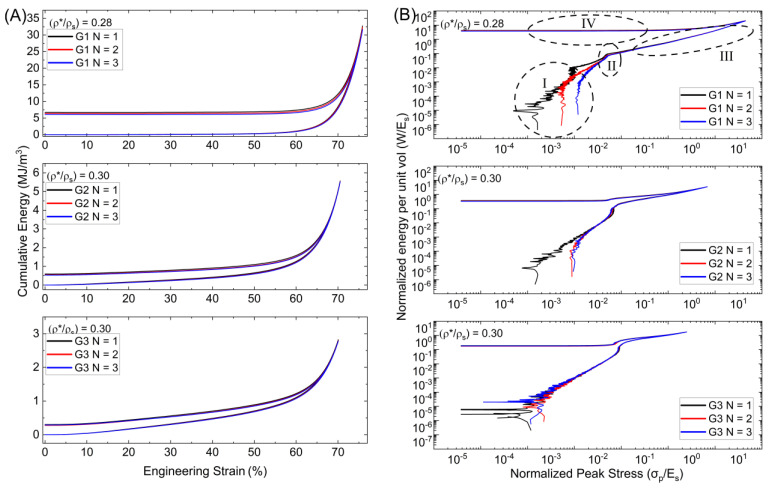
(**A**) Cumulative energy per strain and (**B**) Normalized energy per normalized peak stress for Gyroid lattices.

The Gyroid behaved similarly to the Diamond on *W_v_*-*ε_eng_* curves, with G1 having a greater capacity due to broken walls as is evident in Figure 8. In comparison to the Diamond lattice, G2 and G3 exhibit thinner bands between loading and unloading at greater strain intervals, with comparable recovery behavior. This is most likely due to the cell walls’ orientation regarding the loading direction. Furthermore, whereas both G2 and G3 have a relative density of 0.30, different peak energy values were found. G2 has a cumulative peak energy value of 5.56 MJ/m^3^, while G3 has 2.82 MJ/m^3^, and G1 has 32.4 MJ/m^3^. The slopes present similarly in region I, with the first difference showing in region II. The Gyroid shoulder-point has less prominence than the Diamond lattice. Before region III, there is a small shift in the slope. Here, G2 and G3 present shorter densification regimes than the Diamond before entering the strain recovery phase, excluding G1. The slope then descends gradually from the peak values, with a dip, before attaining a constant rate. Compared to the Diamond lattice, the behavior changes between cycles for G2 and G3, exhibit less variance and weakening. Between cycles N = 1 and N = 2, G2 presents changes, but there is negligible change between cycles N = 2 and N = 3. The Gyroid lattice shows little to no change between loading cycles at the shoulder-points, densification, and strain recovery regimes. In contrast to the Diamond, the Gyroid’s shoulder-points and strain recovery values are far more comparable.

**Figure 9:**
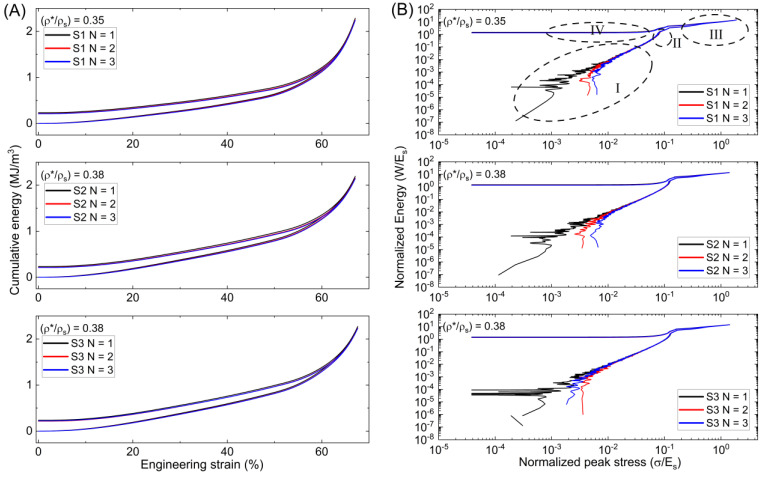
(**A**) Cumulative energy per strain and (**B**) Normalized energy per normalized peak stress for Schwarz lattices.

The results for the Schwarz lattice are presented in Figure 9. The cumulative energy graph vs. strain interval shows a steeper slope and a small band between loading and unloading curves. Peak energy values of 2.29 MJ/m^3^, 2.19 MJ/m^3^, and 2.27 MJ/m^3^ were achieved for S1, S2, and S3, respectively. In the normalized graph, area I has a short slope, resulting in a significantly smaller shoulder-point before entering densification. The Schwarz, like the Gyroid samples, has a brief regime in region III, peaking at a low stress before entering the strain recovery phase of region IV. The initial peak stress variation was greater in the Schwarz. Within Regions II, III, and IV, there is little to no variation between consecutive cycles.

Shorter densification regimes of the Gyroid and Schwarz might be explained by the increasing relative density of the lattices and excess material at the cell wall edges unable to be removed during post-processing. Higher cell wall thickness results in less bending and a collapse of the cell wall, as well as a reduction in the strain at which opposing walls meet. Researchers will benefit from normalized energy relationships when designing functionally porous structures for a particular load and application up to a peak stress. The results of this research show that functionally graded Diamond and Gyroid lattices have considerable mechanical and energy capabilities with the latter exhibiting decreased errors. The blue lines, representing the third cycle (N = 3), tend to show higher normalized energy values across all samples. This could imply some minor form of strain hardening after successive loadings. The closeness of the successive cycles (N = 1, N = 2, N = 3) for each sample demonstrates the materials’ excellent recovery with limited residual deformation. The largest difference amongst the lattices occurs in the plateau and densification regimes, suggesting that the internal architecture of the lattices influences these absorption and dissipation of energy. This might be useful in the design of soft robots, impact protection, actuators, and light weighting. Future study should focus on dynamic loadings, cell topology optimization in multiple planes, variance in strain-rate loadings and dwellings, and effects of temperature variations. Cell wall thickness, cell dimensions, and grade direction could each be investigated. Finally, recent research has largely invested in using rigid materials and cartesian cell mapping, whereas soft materials, cylindrical, and spherical mapping has been largely underreported.

The relative error of prints was calculated using this equation:

(17) RE=(mⅇasured mass−CAD mass)CAD mass×100

**Figure 10:**
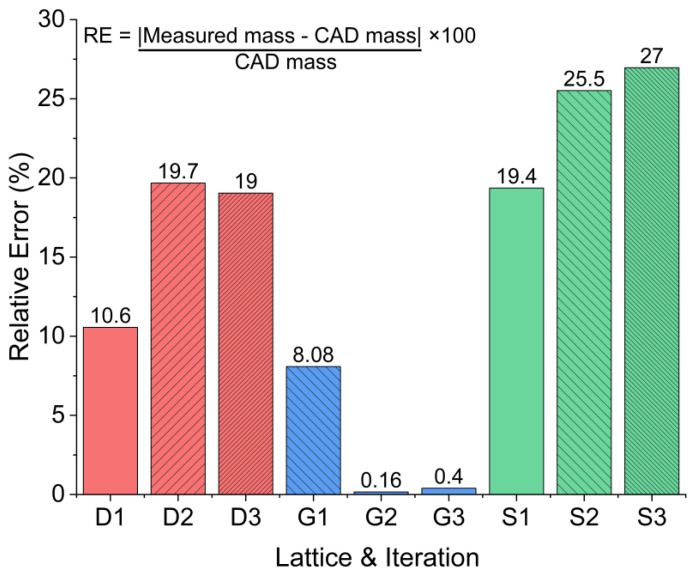
Percent relative error of printed samples determined from mass measurements.

This provides the magnitude of deviation from the CAD design. Calculations were based on the measured mass and the predicted CAD mass of samples for a relative density of 30%. The density of the material, provided by Formlabs, was imported into the material properties of the TPMS structures during the creation of the STL models. The printed models were weighed by calibrated mass scales and the obtained values are shown in Table 1. The Schwarz produced the most relative error shown in Figure 10. This is likely due to extra material in the base and superfluous resin caught within cavities, left over from post-processing. Preform defaults to adding additional material to the base of prints to help ensure printability; the excess resin, here, in this case, was not removed from the base. The Gyroid had the smallest amount of inaccuracy despite this. The Gyroid’s shape, which allows through-channels to be seen on any given side of the structure proves easier to post-process. Similarly, the Diamond has through-channels, but these are situated at the structure corners, making it difficult to drain excess resin from the cell network.

Techniques such as X-ray Computed Tomography (CT), Neutron Tomography (NT), magnetic resonance imaging, Small Angle X-ray Scattering (SAXS) tomography, or digital volume correlation (DVC) in conjunction with micro-CT could be used to enhance error analyses pertaining to manufacturing for the examination of internal deformations under varying load conditions. These methodologies can deliver high-resolution imaging, apt for revealing insights into the intricate microstructural nuances, imperfections, or inconsistencies present within the fabricated lattice.

## 4. Conclusions

In this study, vat polymerization was used to create mathematically modeled, functionally graded Schwarz Primitive, Schoen Gyroid, and Schwarz Diamond structures. During cyclic compressive testing, the mechanical properties, the energy absorption, recovery, and dissipation properties along with the printing accuracy of the lattices were examined, yielding the following research conclusions:

1. Vat polymerization could produce complicated TPMS samples with adequate precision that compare favorably to the intended designs.
2. In the *σ*-*ε* graphs, The Schwarz primitive sample presents a bending-dominated behavior, whereas the Diamond and Gyroid samples show stretching dominance. The Gyroid (adjusted for G1) and Schwarz Primitive lattices came in second and third, respectively, with the graded Diamond lattice producing the greatest modulus and plateau stress values. Energy attributes also somewhat align in this manner.
3. All the tested samples show some resistance to permanent deformation; however, the Schwarz shows larger variation in early strain intervals compared to the Gyroid or Diamond, likely due to the stretching dominance, but remains the most consistent during loading. Because of this, the Diamond and Gyroid sheet TPMS are recommended, as they function well under high strains with desirable energy characteristics. The Schwarz primitive lattice demonstrated the lowest energy properties but was consistent. This could imply that, for applications where consistency is more critical than the total energy amount absorbed, the Schwarz primitive lattice may be preferred. The Schwarz primitive and Gyroid lattices show similar strain recovery rates that differ from the Diamond, likely due to the cell wall orientation. Selecting the optimal lattice structure for specific energy absorption requirements is fundamental in applications like crash-resistant structure or protective gear, where energy dissipation can be lifesaving.
4. A discernible discrepancy exists between the stiffness and plateau stress as forecasted by the Ashby–Gibson models, with the Additively Manufactured (AM) materials markedly surpassing the projected parameters. This divergence could potentially be attributed to the oversimplified assumptions of the models and their consequent neglect of several pivotal factors intrinsic to AM materials. Specifically, considerations pertaining to print orientation, lattice geometry, the incorporation of fillers and additives, the formation of layer boundaries, the meticulous composition of polymers, and cross-linking dynamics are notably absent in the existing predictive frameworks, potentially contributing to the observed discrepancies in performance. This underscores the need for refining and enhancing models to align with advancing engineering technology, especially to bridge the gap between theoretical predictions and real-world performance metrics.
5. Large variations in *σ*-*ε* diagrams reveal variations in material properties for different lattice structures through consecutive loadings. This infers that lattice structures exhibit varied mechanical responses. This results in a demand for engineering design to consider these variations for the structural integrity of prolonged periods. Careful consideration of manufacturing parameters and procedures is crucial for leveraging the full potential of AM technology.
6. Densification and thickness of lattices influences the deformation and collapse of the cell walls and the strain where opposing walls meet. This can impact structural integrity and performance. Designing lattices with appropriate relative density and wall thickness can provide enhanced structural stability and resilience against collapse while removing excess weight. The results obtained can guide the design and manufacturing process, enhancing the performance of samples in real-world applications.

Advancements in the additive manufacturing of lattice structures have revealed limitations in existing theoretical models, demanding the development of refined models and methodologies that can accurately capture the complexities of real-world behaviors of AM materials. Hyper-elastic AM materials demonstrate the capability of recovering from large strains, allowing for the opportunity to capture more strain recovery data. This work highlights the nuances in mechanical performance between the lattices. The variations in mechanical and energy properties among the different lattice structures highlight the requirement for meticulous design and optimization, considering the specific conditions of the end application. The conclusions drawn from this study can significantly influence future research directions, the development of complex structures, and innovative engineering applications, particularly in fields where material performance, precision, and reliability are paramount.

## Figures and Tables

**Table 1:** Mass measurements for relative error calculations.

Sample	Diamond (g)	Gyroid (g)	Schwarz (g)
1	1.382	1.149	1.492
2	1.496	1.248	1.569
3	1.488	1.255	1.587
Theoretical mass (g)	1.25	1.25	1.25
STL file size (MB)	44.65	43.75	29.81
X, Y, Z Cell Size (mm)	11	11	9.25

## Data Availability

The data presented in this study are available in the Supplementary Material section under the name Cyclic Compression Testing in OriginPro© format.

## Supplementary Materials

The following supporting information can be downloaded at: https://www.mdpi.com/article/10.3390/polym15234475/s1. Supplementary Materials are available in the form of OPJU File, readable in Origin Unicode Project. The file contains raw data used to calculate Compressive Mechanical Properties, Energy Absorption Properties and Relative Error, of Hyper-Elastic-Graded Triply Periodic Minimal Surfaces.

## Appendix A

**Table A1:** Mechanical Properties of Elastic 50-A.

	Metric		Imperial		Method
	Green	Post-Cured	Green	Post-Cured	
UTS	1.61 MPa	3.23 MPa	234 psi	468 psi	ASTM D412-06 (A) [37]
σ at 50% Elongation	0.92 MPa	0.94 MPa	133 psi	136 psi	ASTM D412-06 (A)
σ at 100% Elongation	1.54 MPa	1.59 MPa	223 psi	231 psi	ASTM D412-06 (A)
Elongation @ Failure	100%	160%	100%	160%	ASTM D412-06 (A)
Compression set 23 °C for 22 h.	2%	2%	2%	2%	ASTM D395-03 (B) [38]
Compression set for 70 °C for 22 h.	3%	9%	3%	9%	ASTM D395-03 (B)
Tear strength	8.9 kN/m	19.1 kN/m	51 lbf/in	109 lbf/in	ASTM D624-00 [39]
Shore Hardness	40 A	50 A	40 A	50 A	ASTM 2240 [40]
